# Multisystem Inflammatory Syndrome in Children: A Case Report

**DOI:** 10.7759/cureus.72303

**Published:** 2024-10-24

**Authors:** Thryambak Ganapathy, Kirsten Emily M Villagracia, Saharsh S Kuditini, Sorivel Sosa Hilario

**Affiliations:** 1 Medicine, American High School, Fremont, USA; 2 Medicine, Irvington High School, Fremont, USA; 3 Medicine, North Hills Preparatory, Irving, USA; 4 Research Department, Global Health Leaders, Punta Cana, DOM

**Keywords:** antibiotic therapy, anti-parasitic, clinical case report, erythema, mis-c

## Abstract

This case report delves into the case of a patient in the Dominican Republic with multisystem inflammatory syndrome in children (MIS-C). MIS-C is a rare, hyper-inflammatory condition that develops in children as a delayed response to a severe acute respiratory syndrome coronavirus 2 (SARS-CoV-2) infection, typically appearing with general markers of inflammation such as erythema and, in severe cases, cardiorespiratory symptoms. The three-year-old patient discussed in this report presented with signs of inflammation, such as erythema, rashes, and chapped skin, and reported experiencing diarrhea, vomiting, and fever for multiple days. Notably, a concurrent parasitic presence was found in the patient's fecal sample, and antibiotics were heavily used throughout the course of treatment. We explain the merits and drawbacks behind using antibiotic therapy for MIS-C and suggest steps that clinicians and researchers can take in order to minimize the potential misuse of antibiotics. Specifically, we identify that prioritizing tests for concurrent infections or illnesses is imperative in treating MIS-C patients, and we conclude by stating that using blood cultures and coprological examinations in tandem is an effective strategy for this purpose.

## Introduction

Multisystem inflammatory syndrome in children (MIS-C) is a rare hyper-inflammatory disorder that is eminent in children who have been infected with COVID-19, a disease originating from the severe acute respiratory syndrome coronavirus 2 (SARS-CoV-2) strain. MIS-C emerges between two and six weeks after the initial contraction of COVID-19 and is characterized by its severe inflammation of the cardiovascular and respiratory systems. However, it is unclear how MIS-C derives itself from COVID-19, as patients who are both symptomatic and asymptomatic for COVID-19 can develop MIS-C [1]. Regarding epidemiology, studies from regions with multiple racial communities estimate that 40.5% of MIS-C patients are Hispanic/Latino, 33.1% are ethnic African, and 13.2% are non-Hispanic White [2]. Patients with MIS-C are typically between ages 0 and 19, have a fever for at least three days, elevated markers of inflammation, no other causes for inflammation, have evidence of COVID-19, and 2 of the following symptoms: rash hypotension, coronary abnormalities, coagulopathy, and acute gastrointestinal symptoms [1].

A proteomic study of MIS-C revealed that increased expression of phospholipase A2 (PLA2G2A) is a strong biomarker of MIS-C [3]. It found that higher PLA2G2A levels were the most prominent difference between MIS-C patients and the other groups in the study-healthy participants and SARS-CoV-2 patients-indicating that it plays an especially unique role in the progression of MIS-C [3]. Despite the important role of this protein in MIS-C, its mode of action is not fully understood.

One of the most prominent abnormalities that has been consistently observed in MIS-C patients is elevated cytokine levels. MIS-C is characterized by large amounts of interferon-gamma (IFN-γ) [4]. Interferon-gamma, or IFN-γ, plays a crucial role in the immune system's functionality. Upon interaction with IFN-γ receptors on macrophages, it encourages the activation of a pro-inflammatory response through augmented phagocytosis-the process by which macrophages can destroy pathogens-and increased antigen presentation-a mechanism that directs the T lymphocyte immune response in an efficient and targeted manner [5]. 

Studies also commonly point to changes in the prevalence and behavior of T cells as a bioactive factor in MIS-C patients. T cell lymphopenia is frequently observed with MIS-C, and the associated depletion in the body’s white blood cell counts is compensated for by a greater degree of activation in the remaining T cells-evidenced by the more prolific production of CD38 and human leukocyte antigen DR (HLA-DR) seen in MIS-C T cells [6]. CD38 and HLA-DR are proteins that are expressed during T cells’ immune response to antigens, and their expression can serve as reliable indicators of abnormally high T cell activity [7].

These theories are made even more convincing by a case study performed by Lee et al. involving a patient with MIS-C presenting with elevated IFN levels typical of the syndrome [8]. It found that the patient had a shortened variant of the suppressor of cytokine signaling 1 (SOCS1) gene, resulting in a haploinsufficiency of the crucial negative regulator of IFN-γ signaling, from which enhanced interferon and T-cell activity would logically follow [8]. It is entirely plausible that the root cause underlying MIS-C is related to such genetic variants due to the rarity of the syndrome and its prevalence in select racial groups, specifically in Black and Hispanic children [9].

However, this attempt at understanding the pathophysiology of MIS-C is partially undermined by the observation that IL-10 levels are not only elevated in MIS-C patients but also serve as a key distinguishing factor between MIS-C and SARS-CoV-2 due to the fact that COVID-19 patients also exhibit high IFN-γ counts [3]. IL-10, unlike IFN-γ, is an anti-inflammatory cytokine that protects host tissue from inflammation and damage by limiting the intensity of the immune response [10]. In addition, while elevated IFN-γ expression is a crucial aspect of the MIS-C pathophysiology, it has been demonstrated that the severity of the syndrome is negatively correlated with IFN-γ levels [3]. It is, therefore, imperative for future studies to investigate the dynamic between IFN-γ and IL-10 in order to progress towards a more thorough understanding of the etiology behind MIS-C.

Effective treatment options exist to combat the MIS-C inflammatory response. The first line of treatment and most common form of therapy when combating symptoms of MIS-C is intravenous immunoglobulin (IVIG). This form of treatment targets cells called neutrophils, a type of white blood cell, resulting in anti-inflammatory effects on MIS-C [11]. Researchers from the University of California San Diego School of Medicine found that IVIG significantly decreases activated neutrophils, which is a major source of the inflammation enhancer IL-1β [12]. Additionally, glucocorticoids are usually paired with IVIG treatment to reduce the activity of immune cells, therefore diminishing inflammation. Recent studies found that the effectiveness of monotherapy-IVIG alone versus combination therapy-IVIG with glucocorticoids depends on the patient’s responsiveness [13]. 

While IVIG and glucocorticoids are the most common forms of therapy used to treat MIS-C, other treatments, such as therapeutic plasma exchange (TPE) and extracorporeal membrane oxygenation (ECMO), are also used to reduce symptoms of MIS-C. TPE strengthens immune regulation and removes inflammatory mediators by reducing abnormal procoagulant agents and replacing them with the missing coagulation factors [14]. In doing so, TPE targets and helps to eliminate blood clots, which are a significant MIS-C marker. For severe cases of MIS-C, ECMO provides aid for causes of severe pulmonary and cardiac failure. While the exact mechanisms of ECMO in MIS-C are still under investigation, its effectiveness in managing these most severe cases is well documented [15]. Essentially, there is no single treatment plan of choice; rather, clinicians have options that they can explore in order to determine which leads to the most improvement in the patient’s inflammatory symptoms.

## Case presentation

In 2021, a three-year-old girl was brought into the hospital by her grandmother with signs of fever, vomiting, diarrhea, dryness, and erythema. She was reported to be 15 kilograms and mixed race. Prior to her visit to the hospital, she experienced a four-day fever of 39.5°C/103.1°F, vomiting for three days, and diarrhea for two days. She had no siblings, no prior hospitalizations, and had updated immunizations. Upon arriving at the hospital, the child was irritable, complaining, and lost appetite.

A general physical examination of the patient revealed well-known markers of inflammation. She presented with redness of the skin around the eyes, which was interpreted as palpebral erythema due to congestion and distention in the capillaries around the eyes, accompanied by exanthematous lesions and dry rashes in the patient’s palms. The skin around her mouth was markedly chapped-specifically at the corners-and the salivary glands inside the mouth lacked the production level necessary to maintain sufficient moisture in the mouth. Additionally, the patient reported diffuse pain upon palpation around her abdomen. 

Analytics received along with the physical exam indicated that the patient was SARS-CoV-2 immunoglobulin G (IgG) positive and immunoglobulin M (IgM) negative. The patient’s white blood cell (WBC) count was elevated at 22.21 thousand WBCs per microliter. Neutrophil levels were abnormally high at 91%. Monocyte, hematocrit (HCT), hemoglobin, and platelet counts were on the lower end of the spectrum but around the normal range.

**Table 1:** Table comparing laboratory analytics of the patient to the normal ranges expected for a healthy three-year-old. dL: deciliter, mg: milligram, g: gram, mm^3^: cubic millimeter, HCT: hematocrit.

Vital type	Description	Current analytics	Normal analytics (approximately three years old)
Immunoglobulin G (IgG)	Antibodies that are produced one to two weeks after an infection	Positive (abnormal)	600-1700 mg/dL
Immunoglobulin M (IgM)	Antibodies produced against an antigen in the early stages of an infection	Negative (normal)	2-4 years: 37-184 mg/dL
White blood cell (WBC)	Part of the body’s immune response	22,210 WBC/mm^3^ (high)	5,000-19,000 WBC/mm^3^
Hemoglobin (HBC)	Protein in red blood cells that carries oxygen	10.4 g/dL (normal)	9.5-14 g/dL
HCT (red blood cells)	Type of blood cell that is made of bone marrow and contains hemoglobin	30.1% (low)	31-41%
PLT (platelet)	Blood cells that prevent and stop bleeding	150 (normal)	150-450 x 10^9^/L
Neutrophils (NEU)	Type of white blood cells that act as the immune system’s first line of defense	91% (high)	50-70%
Monocytes (MON)	Type of white blood cell that reside in blood and tissues that destroy germs and eliminate infected cells	4.3% (normal)	2-8%

The progression of the patient's case is detailed in Table 2. Upon reception, methylprednisolone was administered intravenously at a dosage of 400 mg in conjunction with 300 mg of IVIG over a 12-hour timespan. Four hours later, after evaluation by the infectious disease department found further evidence of extreme inflammation in peritonsillar plaques and erythematous rashes in the extremities, the patient was given antibiotic medications, vancomycin and ceftriaxone, along with aspirin. Twenty-four hours later, a coprological report revealed the presence of the *B. hominis* parasite in the patient’s fecal sample. Clinicians then added a dose of nitazoxanide to the treatment regimen, along with a second dose of IVIG. After another 24 hours, an examination found that the patient had a D-dimer of 9.49 mg/L, elevated above the normal 0.50 mg/L. The anticoagulant medication enoxaparin was administered through a subcutaneous injection at a dosage of 0.1 mL over 12 hours, along with an increased dosage of aspirin-81 mg per six hours to prevent chest pain or cardiac complications. The patient showed significant improvements over the next 48 hours, with decreases in their edema and respiratory distress. The vancomycin and ceftriaxone treatments were suspended in favor of cefuroxime, another antibiotic, administered orally at a dosage of 5 mL every 12 hours. After 48 hours, the patient was discharged from the infectious disease department with outpatient management of their enoxaparin anticoagulant therapy and then discharged from the cardiology department another 48 hours later under stable conditions.

After exhibiting signs of major improvement, the infectious disease department and the cardiology department discharged the patient under stable conditions shortly after. Ambulatory care was continued in order to manage minimal symptoms, namely, persistent abdominal distention and mild lower limb edema.

**Table 2:** Timeline of case progression and treatment administration. IgG: immunoglobulin G, IgM: immunoglobulin M, WBC: white blood cell, IV: intravenous, DMH: hourly mean diuresis, VO: verbal order, PO: per Os, ESR: erythrocyte sedimentation rate, PCR: polymerase chain reaction, SC: subcutaneous.

Date	Symptoms/findings	Administered treatments	Patient parameters monitored
4/11/21	Four-day fever at 39.5°C, vomiting (three days), no improvement, diarrhea (two days), COVID-19, IgG positive and IgM negative, physical examination: conjunctival infection exanthematous lesions/erythema on palm and plantar level, red mouth mucosa, lab results: high WBC (22.21), high neutrophils (91%), low hemoglobin (10.1), low hematocrit (30.1%), normal platelets (150), normal monocytes (4.3%), and low lymphocytes (4.1%)	Irregular administration of acetaminophen (Tylenol). Oral hydration to counterbalance diarrhea	N/A
4/12/21	6 am: Oliguria, peripheral cyanosis; 10 am: abdominal distention, irritability, loss of appetite, conjunctival injection, white peritonsillar plaques, generalized macular rash, inflammation of extremities, tachycardia, cracked/peeling lips, strawberry tongue, anuria	6 am: Methylprednisolone 400 mg IV, Intravenous immunoglobulin (300 mg, 12 h). 10 am: Vancomycin Ceftriaxone Aspirin (81 mg)	Hypotension (60/40 mm HG), Monitor O_2_ saturation and DMH
4/13/21	Pericarditis, mild systolic dilation, left coronary dilation, mild pericardial effusion. Abdominal distention, hepatomegaly, eyelid edema, ecchymosis in the venipuncture area, palmar and plantar edema, rhonchi and crackles in lungs	The second dose of immunoglobulin only VO administration of nitazoxanide	Requested: D-dimer (9490 ng/ml), PRO-BNP, ferritin (336.6 ng/ml), blood count, PCR, ESR, urine test
4/14/21	24-hour fever	Increased Aspirin dose (81 mg per six hours PO), 0.1 mL enoxaparin SC (every 12 h), discontinued oxygen therapy	N/A
4/15/21	Persistent rhonchi and crackles, mild subcostal retractions	No new treatments were administered	N/A
4/16/21	Clinical improvement: decrease in edema, feverish, no respiratory distress, proper feeding	Suspended: vancomycin, ceftriaxone, ranitidine, dexamethasone. Added: cefuroxime suspension 250 mg (5cc every 12 h PO)	N/A
4/17/21	Decreased abdominal and lower limb edema	Continued prior treatments	N/A
4/18/21	Continued abdominal distention and potential increased severity of inflammation/clotting	Recommended extra 24-hour hospitalization and outpatient anticoagulant therapy	D-dimer: 2180 ng/mL
4/19/21	Stable condition, persistent abdominal distention, mild lower limb edema, and good oral tolerance	Continued prior treatments	N/A
4/20/21	Significant improvement, stable condition	Discharged	D-dimer: 1410 ng/mL

## Discussion

The diagnostic process for this patient involved a series of general examinations, laboratory, and COVID-19 tests. Upon physical examination, apparent signs of inflammation, conjunctival infection, scaly lesions, and erythema were visible. Laboratory test results indicated a high white blood cell count and a high neutrophil percentage, further confirming hyper-inflammation and supporting an MIS-C diagnosis [16]. A challenge that clinicians faced when diagnosing this patient was differentiating between MIS-C and Kawasaki’s Disease (KD), another inflammatory syndrome with similar characteristics to MIS-C. However, specific case details favor one condition over the other. The evidence of past COVID-19 infection or exposure is primarily necessary to make an MIS-C diagnosis. Additionally, while coronary artery dilation can be found in both conditions, it tends to be more common in KD. In contrast, hemodynamic instability, elevated cardiac enzymes, and ventricular dysfunction are typically associated with MIS-C [17]. Elevated white blood cell counts, elevated platelets, and eosinophilia are frequently correlated with KD, while elevated D-dimer, ferritin, and thrombocytopenia are more commonly associated with MIS-C [17]. The patient, in this case, demonstrated some of these clear signs of MIS-C, including heart and gastrointestinal dysfunction, a positive COVID-19 IgG antibody test, erythema, and high levels of ferritin and D-dimer in the bloodstream, enabling clinicians to make an accurate diagnosis and outline an effective treatment plan. 

A significant pattern in the treatment regimen for this MIS-C patient was the heavy usage of antibiotic therapy. The apparent contradiction with this element of the treatment plan is that antibiotics were being used in the absence of any bacterial infection. In MIS-C patients, it has been established that symptoms arise as the result of an extreme pro-inflammatory response to the SARS-CoV-2 virus rather than a particular bacterial or viral infection, with the exception of cases in which the two happen to occur simultaneously. Given that the patient’s clinical examinations did not reveal any pathogenic presence, it can be inferred that no harmful bacterial cells were contributing to the patient’s symptoms, and the modes of action (MOAs) of vancomycin, ceftriaxone, and cefuroxime were, therefore, either irrelevant or insignificant in the patient’s clinical improvements.

Considering that antibiotic medications may not be helpful in treating MIS-C, it is fair to question whether the prevalence of antibiotic therapy in MIS-C treatment plans could create unforeseen problems. According to Baran et al., the overprescription of antibiotics in cases where they are irrelevant contributes to the growing problem of antimicrobial resistance [18]. When patients are given antibiotic treatments, antimicrobial compounds are introduced into the environment and its natural circulations [18]. This exposes bacteria to antibiotic medications, speeding up the process of evolution that allows them to develop resistance mechanisms against various antimicrobial compounds [18]. As a result, many antibiotics are rendered irrelevant against clinically relevant pathogens, such as Staphylococci and Enterococci [18]. 

It is necessary to optimize the usage of antibiotics for MIS-C treatment, but this is made complicated by the fact that they are typically used for immediate relief before an accurate MIS-C diagnosis can be ascertained. As mentioned in the previous section on therapeutic interventions, clinicians began to administer vancomycin and ceftriaxone as a direct response to the infectious disease department’s report on the patient’s peritonsillar plaques and erythema, indicating that this patient’s MIS-C may have initially been mistaken for a *Streptomyces* spp. infection. In other cases, antibiotics have been administered under the impression that a patient suffers from sepsis, a disease that shares symptoms similar to MIS-C [19]. However, patients were typically taken off of antibiotic therapy after making a conclusive MIS-C diagnosis within two days, which would have been a beneficial improvement in the clinicians’ approach to this specific case [19].

Another notable finding, in this case, was the presence of *B. hominis*, a parasite that harms the digestive tract, in the patient’s fecal sample through the coprological report. However, this parasite does not correlate with MIS-C; the patient's vomiting, diarrhea, and abdominal distention aligned with the gastrointestinal effects of *B. hominis* [20]. Failing to diagnose *B. hominis*, as well as any other diseases that are present, could pose a further threat to the patient’s long-term health. Due to the fact that the patient was already rendered immunocompromised due to MIS-C, an additional untreated parasitic infection could detrimentally impact the overall recovery process and worsen pre-existing symptoms in addition to adding new ones. To ensure that this was prevented, the clinicians formulated treatment plans to simultaneously alleviate symptoms from both diseases using different methods of administration (IV, VO) after identifying the diseases and isolating their specific symptoms. By intricately combining the usage of these medications, the clinicians were able to target both the parasitic infection and MIS-C inflammation at once, leading to a gradual decrease in the patient's abdominal distention over the following week. Overall, the discovery of *B. hominis* proved crucial towards the patient's recovery, the administration of disease-specific treatment plans, and the consequential inhibition of MIS-C progression.

## Conclusions

It is extremely important to introduce more information about MIS-C into medical circles. Knowledge about common MIS-C symptoms and clinical presentations would allow clinicians to make definitive and specific diagnoses, therefore avoiding any unnecessary usage of antibiotics and resulting in the exacerbation of the antibiotic resistance crisis. Coprological examinations are a useful tool in the diagnostic procedure for MIS-C. They should be used in conjunction with blood cultures going forward in order to identify and treat concurring illnesses or infections that could develop into severe conditions given MIS-C patients’ immunocompromised state. Tangentially, research on blood cultures with the goal of decreasing the amount of time required to detect a bacterial infection could be vital in improving the specificity and efficacy of diagnoses and treatments-not only for MIS-C, but for a vast range of immune-related conditions.
